# The Venom of Philippine Tarantula (Theraphosidae) Contains Peptides with Pro-Oxidative and Nitrosative-Dependent Cytotoxic Activities against Breast Cancer Cells (MCF-7) *In Vitro*

**DOI:** 10.31557/APJCP.2020.21.8.2423

**Published:** 2020-08

**Authors:** Simon Miguel M Lopez, Jeremey S Aguilar, Jerene Bashia B Fernandez, Angelic Gayle J Lao, Mitzi Rain R Estrella, Mark Kevin P Devanadera, Anna Beatriz R Mayor, Leonardo A Guevarra Jr, Myla R Santiago-Bautista, Olga M Nuñeza, Librado A Santiago

**Affiliations:** 1 *Department of Biochemistry, Faculty of Pharmacy, University of Santo Tomas, Manila, Philippines. *; 2 *Research Center for Natural and Applied Sciences, University of Santo Tomas, Manila Philippines. *; 3 *The Graduate School, University of Santo Tomas Manila Philippines. *; 4 *Department of Biological Sciences, College of Science and Mathematics, Mindanao State University – Iligan Institute of Technology, Iligan City, Philippines. *

**Keywords:** Philippine tarantula, spider venom, cytotoxicity, MCF-7, oxidative stress

## Abstract

**Background:**

Breast cancer is a multifactorial disease that affects women worldwide. Its progression is likely to be executed by oxidative stress wherein elevated levels of reactive oxygen and nitrogen species drive several breast cancer pathologies. Spider venom contains various pharmacological peptides which exhibit selective activity to abnormal expression of ion channels on cancer cell surface which can confer potent anti-cancer activities against this disease.

**Methods:**

Venom was extracted from a Philippine tarantula by electrostimulation and fractionated by reverse phase-high performance liquid chromatography (RP-HPLC). Venom fractions were collected and used for *in vitro* analyses such as cellular toxicity, morphological assessment, and oxidative stress levels.

**Results:**

The fractionation of crude spider venom generated several peaks which were predominantly detected spectrophotometrically and colorimetrically as peptides. Treatment of MCF-7 cell line of selected spider venom peptides induced production of several endogenous radicals such as hydroxyl radicals (•OH), nitric oxide radicals (•NO), superoxide anion radicals (•O^2−^) and lipid peroxides via malondialdehyde (MDA) reaction, which is comparable with the scavenging effects afforded by 400 µg/mL vitamin E and L-cysteine (p<0.05). Concomitantly, the free radicals produced decrease the mitochondrial membrane potential and metabolic activity as detected by rhodamine 123 and tetrazolium dye respectively (p>0.05). This is manifested by cytotoxicity in MCF-7 cells as seen by increase in membrane blebbing, cellular detachment, caspase activity and nuclear fragmentation.

**Conclusion:**

These data suggest that the Philippine tarantula venom contains peptide constituents exhibiting pro-oxidative and nitrosative-dependent cytotoxic activities against MCF-7 cells and can indicate mechanistic insights to further explore its potential application as prooxidants in cancer therapy.

## Introduction

Breast cancer is a heterogeneous and multifactorial disease that affects mainly women and ranks second with respect to major cancer deaths worldwide (Pinkert et al., 2018). Moreover, several epidemiological factors have been associated with breast cancer especially exposure to ionizing radiation, tobacco smoking, alcohol consumption, and hormonal or oral contraceptives which are well-known source of free radicals that could induce several mutations and impairment of the DNA repair system such as double strand breaks (DSBs) that could eventually harbor counterbalance and irregularities to cell proliferation, differentiation, apoptosis and genomic stability (Dexheimer, 2013; Dumitrescu and Cotrala, 2005; Wu et al., 2008). 

Likewise, reactive oxygen/nitrogen species (ROS/RNS) are group of free radicals, ions, and molecules containing singular and unpaired electrons which exhibits highly combative processes in the cells. They are broadly considered into oxygen and nitrogen-free radicals and non-radicals that exhibit normal cellular proliferations, communication, and survival at physiological levels (Kumari et al., 2018). However, internal and external perturbations and insults trigger elevated levels of ROS/RNS that confer oxidative stress (OS) which is one of the known pathological causes of breast cancer progression (Hecht et al., 2016). Interestingly, mitochondria are a prime source of ROS/RNS wherein dysfunction and altered morphology can drive excessive ROS formation that could exacerbate OS that may endure in a self-perpetuating cycle of breast cancer activity (Jezek et al., 2018).

Conversely, spider venom contains various forms of complex active low molecular weight compounds, peptides and proteins which target a different receptor, ion channels, and enzymes (Chen et al., 2018). Consequently, spider venom toxins exhibit several potent cytotoxic properties to several cancer cells through apoptosis modulation, proliferation management, inhibition of enzymatic activities, and cell cycle alterations (Chen et al., 2018). Also, it may exhibit selective activity to abnormal expression of ion channels on cancer cell surface which can confer potent anti-cancer activities (Zhao et al., 2011). 

This is supported by several studies on other spiders such as *Macrothele raven, Lycosa singoriensis, Lachesanatara baevi* and *Psalmopoeus cambridgei *that were found to exhibit anti-tumor activity on erythroleukemia K562 cells, hepatocellular BEL-7402 cells, and HeLa cells which is essential towards potential cancer treatment (Gao et al., 2007; Liu et al., 2012; Dubovskii et al., 2015). Moreover, venom peptides offer several advantages for targeted therapy such as specificity and selectivity to proteins highly expressed in cancer cells (Barrion-Dupo et al, 2016; Garciano et al., 2014). However, potential anti-cancer activities have not been explored yet on venom from Philippine Theraphosidae as these spiders were only studied based on their morphological characteristics and features. Therefore, this study aims to evaluate the oxidative and nitrosative activities of venom peptide constituents from Philippine tarantula as well as their cytotoxic activity against human breast adenocarcinoma (MCF-7) cells for potential therapeutics.

## Materials and Methods

*Spider Collection, Rearing, Maintenance, and Identification*

The spiders were collected from the caves of Bagacay, Surigao in Mindanao, Philippines through hand-grabbing and the use of long forceps. Spiders were maintained in twelve-hour daylight and darkness, fed with *Pycnoscelus striatus* once per week and provided with water *ad libitum* (Estrada-Gomez et al., 2015). Spiders were submitted at the Museum of Natural History, University of the Philippines Los Baños for the morphological characterization and identification of the collected spiders.

*Venom Extraction, Fractionation and Protein Quantification*

The spiders were anesthetized with CO_2_(g) for 5 minutes to ensure immobility. Venom was extracted in the chelicerae at 35V. Spiders were then placed in temporary shelter for recuperation. The crude venom was lyophilized and stored under -20ºC refrigerator (Garciano et al., 2014; Garb, 2014). 

The lyophilized crude venom was diluted with 195 μL of 0.1% trifluoroacetic acid (TFA) in distilled water (dH_2_O) and fractionated using Waters Alliance e2695 RP-HPLC system in a 0-95% gradient elution system with a flow rate of 1 mL/min for 100 minutes using C18 column as stationary phase and 0.1% TFA in dH_2_O and 0.1% TFA in 90% acetonitrile (ACN) as the solvent system mobile phase. The peaks fractions monitored at 215 nm and 280 nm were collected to detect the presence of peptide bonds and aromatic amino acids.

The crude and fractionated venom samples were qualitatively determined for the presence of peptides through bicinchoninic acid (BCA) assay. For this assay, the mixture contained 500 μL of BCA and 10 μL of each sample. The samples were incubated at 60ºC for 30 minutes and read at 562 nm (He, 2011).

*Cell Seeding, Treatment and Lysis*

The breast cancer adenocarcinoma, MCF-7 cell line from the American Type Culture Collection (ATCC), passage number- P12, were seeded followed by incubation at 37ºC in a humidified incubator with 5% CO_2_ for 24 hours. Venom fractions (50 μL of fractions 1, 3, 10, 12, 14, and 19) and controls (400 μg/mL vitamin E and 400 μg/mL of L-cysteine) were administered in the cell culture flasks containing Dulbecco’s Modified Eagle Media (DMEM) and incubated for 24 hours. The medium was removed, treated with trypsin, centrifuged at 5,000 rpm, and treated with 6 mL lysis buffer to obtain the lysate.

*Detection of Cellular Stress Levels*

Production of hydroxyl radical (•OH) was conducted according to the methodology of Santiago and Mayor (2014) with some modifications. In every treatment group, 10 μL of 0.1 mM EDTA, 1 μL of 0.1 mM FeCl_3_, 10 μL of 2 mM H_2_O_2_, 36 μL of 3 mM deoxyribose, 33 μL of 20 mM phosphate pH 7.4, and 100 μL of cell lysate were mixed and incubated for 30 minutes at 37ºC. Afterwards, the samples were added with 50 μL of 5% (w/v) trichloroacetic acid (TCA) and 50 μL of thiobarbituric acid (TBA) and were incubated for 30 minutes in 37ºC. The absorbance of each solution was read at 532 nm.

The production of nitric oxide radical (NO•) was conducted based on the methodology of Santiago et al., (2016) with some modifications. In every treatment group, 400 μL of 10 mM SNP, 100 μL of PBS and 100 μL of cell lysate were combined and incubated for 25 ºC for 150 minutes. Afterwards, an aliquot of 50 μL was combined with 100 μL of Griess reagent containing 1% (w/v) sulfanilamide, 2% (v/v) H_3_PO_4_ and 0.1% (w/v) NED-HCl. The absorbance of each solution was read at 546 nm.

Formation of superoxide anion radical (•O^2-^) assay was conducted according to the methodology of Santiago and Mayor (2014) with some modifications. In every treatment group, 100 μL of cell lysate was added with 50 μL of 73 μM of NADH, 156 μM of NBT and 60 μM of PMS and incubated for 5 minutes at 25 ºC. The absorbance of each solution was read at 560 nm.

The detection of reduced glutathione (GSH) assay was done according to the methodology of Arimado and Santiago (2015) with some modifications. In every treatment group, 100 μL of cell lysate was added with 270 μL of 50 mM phosphate buffer pH 7.0 and 20 μL of 4.5 mM DTNB. The absorbance of each solution was read at 412 nm.

The lipid peroxidation assay was conducted according to the methodology of Acosta et al., (2013) with some modifications. In every treatment group, 2 mL of 1:1 0.15% (w/v) TCA and 0.25 M HCl were added in 100 μL cell lysate and incubated in water bath for 15 minutes at 100ºC followed by cooling at 25ºC. Afterwards, the absorbance was read at 535nm.

*Mitochondrial Membrane Potential and Impairment Assay*

The mitochondrial membrane potential assay was conducted according to the methodology of Sakamuru et al. (2012) with modifications. The microtubes containing 1.5 mL of cell lysate were centrifuged at 2,000 rpm for 10 minutes at 4ºC. The supernatants were further centrifuged at 10,000 rpm for 10 minutes at 4ºC. Afterwards, the precipitate was mixed with 100 μL of rhodamine dye and 20 μL of the samples and was placed in a 96 microwell plate. The absorbance was read at 507 nm. 

The mitochondrial impairment assay was based on the reduction of the 3-(4,5-dimethylthiazol-2-yl)-2,5-diphenyltetrazolium bromide (MTT) dye into its formazan product via enzymatic reaction of mitochondrial NAD(P)H-dependent cellular oxidoreductases. The media from each treatment group of MCF-7 cell lines were removed and 20 μL of 1 mg/mL concentration MTT dye was added into each well containing the untreated and treated cells. The microplate containing the cells was incubated for 4 hours at 37ºC humidified incubator with 5% CO_2_(g). After incubation, 200 μL of 0.1% DMSO was added to each well and the absorbance of each solution was read at 570nm.

*Cellular Morphology Analysis*

The morphological features of the treated and untreated MCF-7 cell lines were first assessed using bright-field and fluorescence microscopy to analyze their cytoplasmic and nuclear characteristics. Afterwards, the activity of caspase 3/7 was determined using 20 μL of 5 μM of caspase 3/7 green detection reagent. Subsequently, the cells were treated with Hoescht 33342 dye in a complete medium for 30 minutes at 37ºC in the dark.

*Statistical Analysis*

Microsoft Excel version 2007, pH stat and GraphPad Prism 6 were used for the different statistical analysis throughout the study. Each of the assay was conducted in triplicates, the mean and standard error mean (SEM) were obtained from each assay. One-way analysis of variance (ANOVA) was employed to determine the significance between the treated and untreated groups wherein p-values <0.05 were considered significant at 95% confidence interval.

## Results

*Chromatographic Profile and Peptide Determination of the Philippine Tarantula Venom*

**Figure 1:**
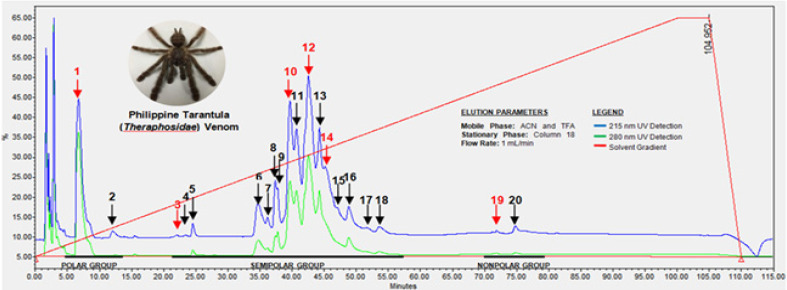
RP-HPLC Profile of Crude Spider Venom from Philippine Tarantula (Theraphosidae). Arrows indicate the peaks which were collected as fractions while the red arrows depict fractions used for oxidative and cytotoxic assays

The chromatographic profile of the crude spider venom fractionated by C18 RP-HPLC using ACN and TFA as solvent system via gradient elution for 100 minutes generated 20 distinct and overlapping peaks monitored and detected at 215 and 280 nm as indicated in Figure 1. The peaks represent different compounds which predominantly contain different forms of peptides as indicated by their retention times. In addition, the elution behavior of each fractional constituent is influenced by the composition of the solvent system which was semi-polar in nature and affected the elution profile of spider venom. In addition, the peak intensities indicate the differences in levels of peptides in the spider venom which are chiefly present in the semi-polar region of the chromatogram.

In addition, qualitative results presented that the crude spider venom and its corresponding fractional constituents produced intense and faint purple colorations respectively which can deduce due to different intensities of the concentration of peptides in crude and fractionated venom respectively.

*Detection of Intracellular Reactive Oxygen and Nitrogen Species Production*

**Figure 2:**
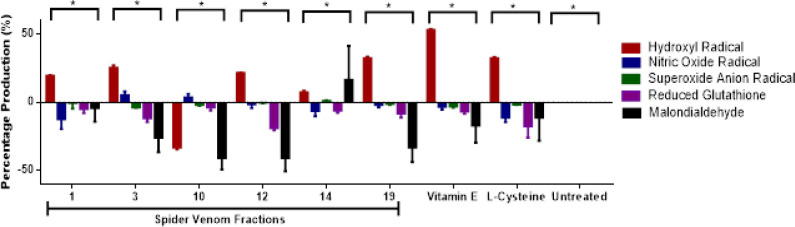
Production of Various Endogenous Radicals after Treatment of Selected Spider Venom Fractions in Comparison with the Controls Vitamin E, L-cysteine and Untreated Group. Results are expressed as mean ± SE (bars), (Note: *p<0.05 significant differences against the negative control) (n=3).

Fractions 1, 3, 10, 12, 14, and 19 were randomly selected from the pool of fractions from the crude spider venom for the biological assay against MCF-7 cell lines alongside with positive controls 400 μg/mL of vitamin E, L-cysteine which is the gold standard for oxidative assays under hydrophobic conditions and the amino acid to exhibit antioxidative properties in the cell lysate, respectively and negative control which is the untreated cell line group as indicated in Figure 2.

Based on the results, the cells treated with venom fractions 1, 3, 12, 14 and 19 stimulated •OH formation while fractions 3 and 10 induced NO• production in MCF-7 cell lines. Additionally, the cells treated with fraction 14 also detected •O^2- ^and MDA. All of the ROS/RNS observed in venom-treated MCF7 cells are comparable with the 400 μg/mL of vitamin E and 400 μg/mL of L-cysteine treated cells which only induced •OH production while none of the oxidative and nitrosative species are observed in comparison with the untreated cell lines

*Mitochondrial Membrane Potential and Impairment Assay*

**Figure 3:**
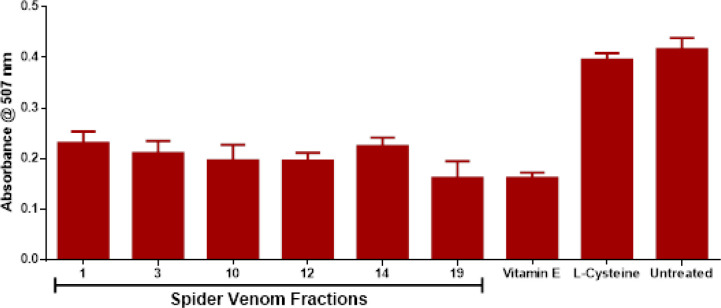
Effect of Selected Venom Fractions and Positive Controls to Mitochondrial Membrane Potential (MMP). Results are expressed as mean ± SE (bars), (Note: *p<0.05 significant differences against the negative control) (n=3).

**Figure 4:**
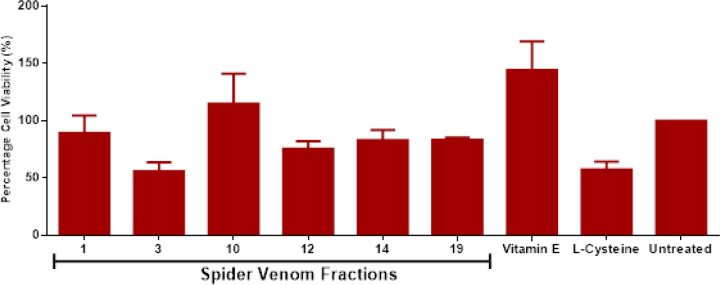
Effect of Selected Venom Fractions and Positive Controls to Mitochondrial Metabolic Activity and Cell Viability. Results are expressed as mean ± SE (bars), (Note: *p<0.05 significant differences against the negative control) (n=3)

The same venom fractions and controls were also evaluated for their effects on MMP since increase in mitochondrial dysfunctions would drive oxidative and nitrosative stress towards cellular apoptosis as represented in Figure 3. Based from the results, significant reduction in rhodamine dye absorbance was observed in the entire treatment group except for cysteine in comparison with the untreated group. This is highly attributed to the loss of protons (H^+^) between the intermembrane space of the mitochondria which could drive increase in mitochondrial activity towards cellular stress. Consequently, the increase in cellular stress drives decrease in cell viability in all of the treated groups except vitamin E in comparison with the untreated group which is determined via mitochondrial metabolic activity through micro-culture tetrazolium (MTT) assay in Figure 4. Hence, it is suggested that the increase in mitochondrial dysfunction and metabolic activity via reduction of MMP are contributory factors to decrease in cell viability in venom-treated MCF-7 cell line groups.

*Cellular Morphology Analysis*

**Figure 5:**
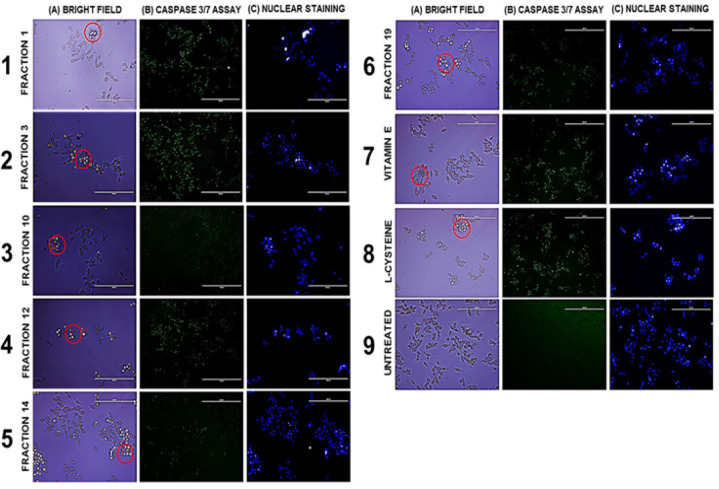
(A) Bright field observation (B) Caspase 3/7 Assay and (C) Hoescht 33342 staining cells treated with selected venom fractions and controls 1) Fraction 1, 2) Fraction 3, 3) Fraction 10, 4) Fraction 12, 5) Fraction 14, 6) Fraction 19, 7) 400 μg/mL Vitamin E, 8) 400 μg/mL L-cysteine and 9) Untreated cell group. Magnification Scale: Bright-field observation and Hoescht 33342 Staining= 200 μm, Caspase 3/7 Staining= 400 μm. Red circles in the bright-field visualization highlight the cell blebs and bodies formed as a result of cytotoxicity of spider venom toxins in MCF-7 cancer cell lines. Green and blue fluorescence indicate the activity of caspases and DNA fragmentation levels as a product of cell death processing

Imaging results of all the treated groups have distinct morphological characteristics with one another as supported by bright-field observation in comparison with the untreated cells as indicated in Figure 5. Generally, distinct evidence of membrane blebbing, cell shrinkage, aggregation and complete detachment from cells in comparison with the untreated group may indicate characteristics of apoptosis as indicated in Figure 5A1-A9.

Likewise, the cells were assessed for their apoptotic activities through executioner caspase 3/7 assay as indicated by figures 5B1-B9. Based from the results, strong and green fluorescent signals were observed on fractions 1, 3 and 12 which were comparable with vitamin E and L-cysteine (400 μg/mL) while weak signals were observed on fractions 14 and 19 which may suggest caspase-dependent apoptosis. On the other hand, no signal was observed in fraction 10 which is similar to the untreated cell group. 

Moreover, evaluation of nuclear morphology was conducted using Hoescht 33342 dye to assess the number of viable cells and morphological features of apoptotic cells through fluorescence labelling at its nuclear DNA as indicated in Figures 5C1-C9. Based from the results, strong signals were observed on venom-treated cells which is comparable with 400 μg/mL of vitamin E and 400 μg/mL of L-cysteine treated group which might indicate reduction of cells via fragmentation, degradation and condensation of nuclear DNA as part of the apoptotic process in comparison with the untreated cells. In addition, distinct round shape morphology was observed in the nucleus of untreated cells which indicates non-apoptotic and healthy features of cell whereas scattered and indistinguishable nuclear morphology were observed on treated cells which may suggest cellular apoptotic processing.

## Discussion

*Philippine Tarantula Venom Predominantly Contains Peptides*

Spider venom is a water-soluble mixture of compounds present in specialized structures that contains multitude of bioactive chemicals which are primarily used by spiders for defense against predators or natural enemies and offense during subduing and killing their prey. These compounds can occur in various forms ranging from small, simple acids, intermediate-sized chemicals and large, complex macromolecules which are made up to at least 35 amino acid residues (Schimdt, 2019). The biological functions of spider venom range from antimicrobial, analgesic, hemaglutinic, neurotoxic, necrotic and cytotoxic activities (Wu et al., 2008). Furthermore, their peptide toxins produce lethal effects on tumor cells by targeting several ion channels and receptors which regulate cell cycle activities, activate caspase pathway or inactivating mitochondria which provide vital insights to explore its potential chemotherapeutic effects against breast cancer (Wu et al., 2008).

The chromatographic profile of the Philippine tarantula venom from Theraphosidae provided insights on the possible components present on its venom. According to the UV spectra library, the wavelength range for peptide bond detection is at 210-220 nm while the aromatic amino acids are detected at 280 nm. 

Based from the chromatogram, polar groups include peaks 1 and 2, semi-polar groups involve peaks 3 to 18 and non-polar groups comprise peaks 19 and 20 as supported by the study of Escoubas et al. (1997). Likewise, the study gives a glimpse of the possible components of spider venom which are polar low molecular compounds, semi-polar peptides ranging from 2000-8000 Da and non-polar proteins and enzymes greater than 8000 Da (Escoubas et al., 1997; Gao et al., 2007). Hence, it can be surmised that peaks 1 and 2 of the polar region are composed of low molecular weight compounds, multiple peaks from peaks 3-18 of the semi-polar region constitute the peptides while peaks 19 and 20 are made up of high molecular weight compounds (Gao et al., 2007).

*Venom Peptides Induce Oxidative and Nitrosative Stress-Mediated Cellular Response on MCF-7 Cell Lines and Exhibit Mitochondrial and Caspase 7-Dependent Apoptosis*

Several studies have supported that spider venom peptides have demonstrated cytotoxicity either through inhibiting cell growth, impairment of cell membrane, induction of apoptosis and decreased cell proliferation (Dubovskii et al., 2015; Liu et al., 2012). The cytotoxic activities of spider venom peptides have been recorded on *P. cambridgei, L. singoriensis, L. tarabaevi,* and *M. raven *in various cell lines through various mechanisms of action (Wu et al., 2008). In the case of Philippine tarantula venom, peptide fractions triggered ROS/RNS in MCF-7 cell lines specifically •OH, NO•, •O^2-^ , and MDA while GSH production was not observed in all the fractions suggesting an oxidative and nitrosative-stress inducing property of the fractions from the crude venom. Consequently, these free radicals have induced decrease in mitochondrial membrane potential (MMP) and mitochondrial NAD(P)H-oxidoreductase activities as detected which resulted in decreased cell viability of MCF-7 suggesting oxidative and nitrosative-dependent cytotoxic capacity of the spider venom fractions (Wu et al., 2008). Several studies from analyzing the morphology of MCF-7 cells suggest it possesses cellular or organellar membrane receptors and ion channels which are highly expressed in cancer cells wherein the spider venom peptides may either interact intracellularly or extracellularly to exhibit cytotoxicity (Waks and Winer, 2019; Ma et al., 2017). 

Peptides purified from several animal venoms offer several advantages for treatment of different diseases such as small size, uncomplicated synthesis, cell penetrating activity, minimal drug-drug interaction, higher activity, specificity, and biological and chemical diversity. Moreover, they have also minimal side effects and immunogenicity since their amino acid constituents can be recycled for cellular biosynthesis of other proteins (Marqus et al., 2017).

On the other hand, mitochondria are the predominant organelle for numerous bioenergetic processes that maintain metabolic activities within the cell, it can also be subjected to several perturbations which drive oxidoreductases to impair redox homeostasis through endogenous free radical production and facilitate OS wherein it can impact several transformations of ROS/RNS to elicit cytotoxic effect on cells (Jezek et al., 2018). One of the known mechanisms of ROS/RNS production is superoxide anion radical (•O^2-^) generation via NADPH oxidoreductase through one-electron reduction of molecular oxygen (O_2_) and downregulation of antioxidants. This can be converted into hydrogen peroxide (H_2_O_2_) by superoxide dismutase (SOD) which can be transformed into its free radical product hydroxyl radical (•OH) through Haber-Weiss or Fenton reaction. The reaction of •OH with lipids within the cell initiates a cascade of lipid peroxidation reactions which generally involve oxidation of several fatty acids which are further decomposed to several lipid peroxide products such as malondialdehyde (MDA) (Gaschler and Stockwell, 2017). These toxic forms of oxygen and nitrogen species are converted into their nontoxic forms through oxidation of reduced glutathione (GSH) which is not evident of the intracellular ROS/RNS detection in treated cells (Liguori et al., 2018). In comparison with vitamin E and L-cysteine their oxidative activities as demonstrated by the intracellular •OH production may suggest a confounding factor on high concentration and dosage applied on MCF-7 cancer cells which are supported by several studies (Pearson et al., 2006).

The cumulative effects from ROS/RNS activities created an impact towards mitochondrial dysfunction particularly loss of mitochondrial membrane potential (MMP) and decreased respiration and oxidative phosphorylation (OXPHOS) via reduction of oxidoreductases activity. MMP forms the transmembrane potential of hydrogen ions (H^+^) which makes the ATP alongside with proton gradient (∆pH) and is generated by protons pumps. However, oxidative and nitrosative stress perturbations resulting to mitochondrial homeostasis through proton gradient depolarization from the intermembrane space which can occur either import or export of protons in the matrix or in the cytosol respectively (Zorova et al., 2018). Furthermore, the loss of MMP leads to unwanted loss of mitochondrial and cell viability as which might result to mitochondrial outer membrane permeabilization (MOMP) wherein pro-apoptotic factors such as cytochrome C, apoptosis-induced factor (AIF) and caspases are released in the cytosol to initiate programmed cell death.

From this standpoint, the OS and mitochondrial-driven cytotoxicity of the spider venom peptides presented notable morphologies in cells which are probable signs of MCF-7 cell integrity, viability or death. Visual observation on bright-field images have noted cell shrinkage and detachment of MCF-7 cells. Moreover, extensive plasma membrane blebbing followed by formation of apoptotic-like bodies through budding process are seen in the MCF-7 treated groups. Noteworthy, caspases are categorized into initiator and executioner enzymes which cleave several substances and facilitates morphological and biochemical changes in cells, one of which is DNA breakage. This is manifested through fragmentation of the genomic DNA of the cells from the compounding effects upstream which is initiated by oxidative, mitochondrial and caspase driven cytotoxicity of the spider venom from Philippine tarantula (Van Opdenbosch and Lamkanfi, 2019). This oxidative and mitochondrial-driven cytotoxicity of spider venom peptides may indicate potential mechanistic insight that ROS/RNS elevation above certain level may induce death in cancer cells which could be an attractive strategy for purported breast cancer treatment applications. This represents new insights on the potential role of prooxidants in cancer therapy.
